# One-Step Fabrication of Dual Responsive Lignin Coated Fe_3_O_4_ Nanoparticles for Efficient Removal of Cationic and Anionic Dyes

**DOI:** 10.3390/nano8030162

**Published:** 2018-03-14

**Authors:** Xingang Li, Youyi He, Hong Sui, Lin He

**Affiliations:** 1 School of Chemical Engineering and Technology, Tianjin University, Tianjin 300072, China; lxg@tju.edu.cn (X.L.); youyihe@tju.edu.cn (Y.H.); suihong@tju.edu.cn (H.S.); 2 National Engineering Research Centre for Distillation Technology, Tianjin 300072, China; 3 Collaborative Innovation Center of Chemical Science and Engineering, Tianjin 300072, China

**Keywords:** lignin, magnetic nanoparticles, one-step fabrication, dye removal, water treatment

## Abstract

A new, simple one-step approach has been developed to synthesize lignin and lignin amine coated Fe_3_O_4_ nanoparticles. These nanoparticles (lignin magnetic nanoparticles (LMNPs) and lignin amine magnetic nanoparticles (LAMNPs)) are found to possess not only magnetic response but also pH-dependent adsorption behavior. Results show that the combination of lignin with nanoparticles increased the adsorption capacities 2–5 times higher than other traditional single lignin based adsorbents (211.42 mg/g for methylene blue (MB) by LMNPs and 176.49 mg/g for acid scarlet GR (AS-GR) by LAMNPs). Meanwhile, by simply adjusting the pH, the dye-loaded adsorbents can be regenerated to recycle both adsorbents and dyes with a desorption efficiency up to 90%. Mechanistic study shows that dye structure and surface charges of adsorbents play the most important part in adsorption where dyes interact with the adsorbent surface via π–π stacking and electrostatic attraction interactions. The efficient fabrication method, eco-friendly reactant, quick magnetic separation, high adsorption and desorption efficiency suggest this novel type of nano-adsorbents to be promising materials for efficient dye pollutant removal and recovery.

## 1. Introduction

Dyes are challenging to remove because of their stable and unreactive properties [1]. Up to now, various technologies have been developed to deal with dye contaminants in wastewater, such as adsorption, membrane filtration, catalytic degradation, flocculation, advanced oxidation process, etc. [2]. Among these methods, adsorption is regarded as a promising strategy due to its high efficiency, economic feasibility and simplicity of operation. Thus, great efforts have been dedicated to synthesize advanced materials for the removal of dyes from wastewater [3,4,5]. 

Lignin, one of the main constituents of lignocellulsoic biomass, is the second most abundant biopolymer on earth [6]. Recently, special attention has been focused on investigating the adsorption behaviors of lignin materials, prompting the possibility of converting this renewable source into adsorbents. Some published work show that lignin possesses adsorption property to heavy metal ions and dyes in waste water, indicating the potential of lignin as adsorbents. Accordingly, during the past decade, great efforts have been made to develop lignin based adsorption materials [7,8,9,10,11]. However, the adsorption capacity of lignin to dyes is still low, even less than 20 mg/g [9,12]. Furthermore, it is a challenge to remove the lignin from the aqueous solution after its adsorption process, especially when the lignin materials exhibit relatively good dispersion behavior in water. 

To enhance the adsorption capacity of the adsorbent, many efforts are made to increase the surface-area-to-volume ratio. In other words, the size of the adsorbent materials should be as small as possible, i.e., nanoscale. Lignin nanoparticles (LNPs, including capsules and tubes) possess huge surface area. They are prepared by anti-solvent precipitation [12,13], self-assembly [14,15,16], interfacial polymerization/crosslinking [17], using nanopore alumina membranes as a template [18], mechanical shearing [19] and sonication [20]. LNPs exhibit higher anti-oxidant activity and enhance material blending. However, the obtained LNP shape is found to be irregular and uncontrollable with low mechanical strength [14]. Additionally, most are unstable in water. They would be soluble under alkali condition, leading to the difficulty in separation from water solution. The involvement of hazardous solvents, such as ethylene glycol (EG), acetone, tetrahydrofuran (THF), etc. during the complex manufacturing processes of LNPs also hinders their applications.

With relatively small size and good dispersion behavior, the nano-adsorbents in water are hard to separate. To intensify the separation efficiency of nano-adsorbents, magnetic nanoparticles (MNPs) were composited into the nano-adsorbents [21,22,23,24]. Cheng et al. [25] prepared graphene oxide enhanced magnetic composite gels (mGO/PVA CGs) which showed remarkably enhanced adsorption capacity for two kinds of cationic dyes with rapid magnetic separation. With the ultra large aromatic structure of GO, the maximum adsorption capacity is up to 231.12 mg/g for methylene blue (MB), which is 2–4 times higher than other magnetic GO composite. Unfortunately, this adsorbent is found to perform poorly in adsorbing anionic dyes (only 20–30 mg/g for methyl orange) and the price of the GO is rather expensive. Li et al. [12] prepared magnetic lignin-based hollow microspheres (MLS) through mixing the lignin hollow microspheres together with the magnetic particles in THF/water solution. Their results show that the generated MLS exhibit adsorption capacities of 31.23 mg/g for methylene blue (MB) and 17.69 mg/g for Rhodamine B (RB), respectively, which are similar to simple lignin’s adsorption capacities. Although MLS can be separated from water solution, the hydrophobic property of the reactant organosolv lignin and microscale of the MLS make its adsorption capacity relatively low. 

Accordingly, it is our aspiration to develop a new method to manufacture LNPs based on coating lignin onto other nanoscale particle templates [26]. Herein, lignin and lignin derivative are anchored on the surface of Fe_3_O_4_ nanoparticles efficiently and simply to produce magnetic nanoscale adsorbent with huge active surface area and magnetic response. To address the challenge, firstly, we introduced a one-step approach to prepare lignin and lignin derivative coated magnetic nanoparticles (LMNPs and d-LMNPs). Compared with the traditional methods mentioned above, this approach shows remarkably combined merits: (1) this method can transfer lignin and iron salts to nanoscale adsorbent without any organic solvent addition or further chemical treatment; (2) the method is much simpler with higher yield; and (3) the LMNPs and d-LMNPs not only show a strong magnetic response but also exhibited a nearly 2–5 times higher adsorption capacity than other lignin based adsorbents. We also try to make this nanoparticles recyclable for green use. Finally, revealing the mechanisms of the adsorption and desorption behavior of LMNPs and d-LMNPs is also our purpose.

## 2. Materials and Methods 

### 2.1. Materials

Chemicals, including ferrous chloride tetrahydrate (FeCl_2_·4H_2_O, AR), ferric chloride hexahydrate (FeCl_3_·6H_2_O, AR), ammonium hydroxide (AR, 25 wt % in water), sodium hydroxide (NaOH, AR), hydrochloric acid (HCl, AR, 37 wt % in water), and formaldehyde (HCHO, AR, 37 wt %), were purchased from Jiangtian Chemical Co., Ltd. (Tianjin, China). Alkaline lignin was purchased from TCI development Co., Ltd. (Shanghai, China). Diethylenetriamine (DETA, C_4_H_13_N_3_, AR) was purchased from Energy Chemical Co., Ltd. (Shanghai, China). Methylene blue (MB) and Acid Scarlet GR (AS-GR) were provided by Heowns biochemical technology Co., Ltd. (Tianjin, China). The structures of MB and AS-GR are shown in Table 1. Sodium hydroxide, hydrochloric acid and ammonium hydroxide were diluted to suitable concentration for use. All chemicals were used without further purification.

### 2.2. Preparation of Lignin Amine (LA)

**Figure 1:**
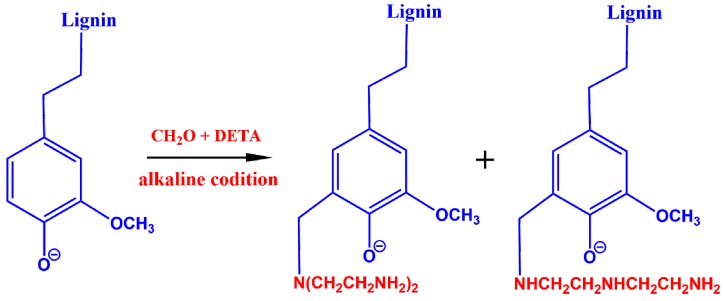
Synthesis of lignin amine.

The alkaline lignin (20 g) was dissolved in NaOH solution (100 mL) at pH 12 and 50 °C. The dissolved lignin was further reacted with DETA (12 g) at 90 °C. Successively, 18 mL of HCHO solution (37 wt %) were added to the mixture dropwise. This mixture was kept reacting for 4 h before the lignin amine was precipitated by HCl (37 wt %) solution [27,28,29]. The brown precipitate was collected by suction filtration, followed by washing with 1 M HCl solution and freeze-dried under vacuum. Consequently, the DETA side chains were grafted to the lignin at the ortho position of phenolic hydroxyl groups, obtaining the lignin amine, shown in Figure 1. 

### 2.3. Preparation of Lignin and Lignin Amine Coated Fe_3_O_4_ Nanoparticles

**Figure 2:**
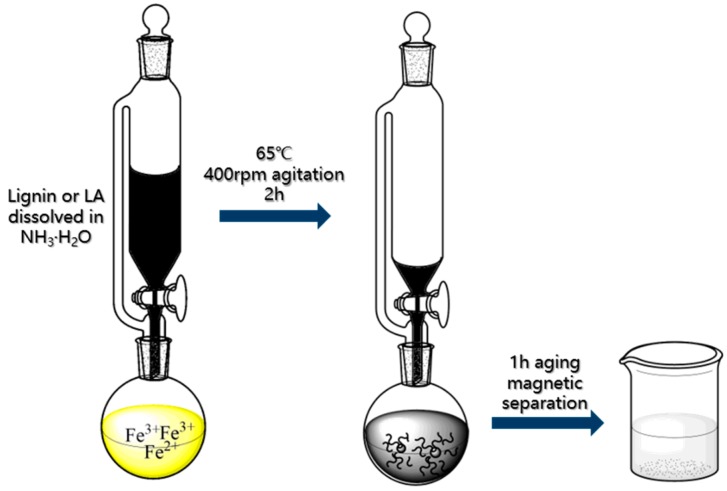
One-step fabrication of lignin coated Fe_3_O_4_ nanoparticles (LMNPs) and lignin amine coated Fe_3_O_4_ nanoparticles (LAMNPs).

The above lignin and the synthesized lignin amine were further used as coating agent on the magnetic nanoparticles. Herein, inspired from the co-precipitation method [26,30,31], we developed a one-step reaction to synthesize the lignin grafted magnetic nanoparticles (LMNPs) and the lignin amine grafted magnetic nanoparticles (LAMNPs), as shown in Figure 2. The detailed procedures are given as follows: FeCl_3_·6H_2_O (0.54 g) and FeCl_2_·4H_2_O (0.2 g) were added together into 100 mL water in a three-neck flask with protection of nitrogen atmosphere. The solution was stirred at 400 rpm and 65 °C for 10 min. Then, 10 mL NH_3_·H_2_O solution (5 wt %) with dissolved lignin or lignin amine were added dropwise by a pressure-equalizing dropping funnel. To keep the pH above 10 during the whole process, 30 mL of NH_3_·H_2_O solution (5 wt %) were added to the flask dropwise. The sample was kept reacting at 65 °C for 120 min. The obtained black solution was cooled down under room temperature and aged for 60 min. Finally, the LAMNPs were separated by magnet and washed by deionized water three times. 

For LMNPs with higher lignin dosage, the solution was quite stable and cannot be separated by magnetic field easily. The pH of the solution was firstly adjusted to 2–4 and separated via magnetic field. After decanting the washing solution, the NPs were dispersed into water and dried by a vacuum freeze dryer. The prepared LMNPs and LAMNPs were about 0.30 g and used for dye adsorption without further treatment.

### 2.4. Adsorption and Desorption of Different Dyes 

The adsorption capacities of LMNPs and LAMNPs under different pH conditions were evaluated by the adsorption of two different dyes: methylene blue (MB) and acid scarlet GR (AS-GR) (Table 1). Briefly, dyes are firstly dissolved into deionized water at the concentration of 500 mg/L. This solution was further diluted to 50 mg/L solution at designed pH values. Subsequently, 20 mg LMNPs or LAMNPs were added to above diluted dyes solution (20 mL). This mixture was sealed and shaken for 24 h at 25 °C until the adsorption reached equilibrium. After the adsorption reached equilibrium, the nanoparticles were separated by a permanent magnet. With similar procedure, the adsorption isothermals of MB and AS-GR on the LMNPs and LAMNPs were tested at pH 10 and 2, respectively, at different concentrations (from 50 to 300 mg/L). For the kinetic studies, 50 mg LMNPs or LAMNPs were dispersed into 80 mL MB or AS-GR solutions (100 mg/L). The pH of the suspension was adjusted to 10 and 2 for MB and AS-GR solutions, respectively. The samples of the solution were withdrawn at appropriate time intervals.

Regeneration of the adsorbent is the key point of chemical or environmental processes. Herein, to reuse the LMNPs or LAMNPs, the LMNPs or LAMNPs (20 mg) adsorbed by dyes at maximum adsorption capacity were dispersed into 10 mL aqueous solution. The desorption was conducted at pH 12 and pH 2 for 3 or 12 h for AS-GR and MB, respectively, under 25 °C water bath oscillation.

The concentrations of dyes in the supernatant were determined using UV-vis spectrophotometer at wavelength of 622 nm and 508 nm for MB and AS-GR, respectively. The adsorption removal efficiency (*R_e_*) adsorption capacity (*q_e_*) and desorption efficiency (*D_e_*) were calculated by Equations (1)–(3), respectively:

(1) $$R_{e}=\frac{C_{0}-C_{e}}{C_{e}}\times 100\%$$

(2) $$q_{e}=\frac{\left(C_{0}-C_{e}\right)\times V}{m}$$

(3) $$D_{e}=\frac{C_{D}V_{D}}{q_{D}m_{D}}\times 100\%$$

where *C*_0_ (mg/L) and *C_e_*(mg/L) are the initial concentration and the equilibrium concentration after adsorption, respectively; *m* (mg) and *V* (L) stand for the dosage of NPs and the total volume of the solution, respectively; *C_D_* (mg/L) and *V_D_* (L) represent the dye concentration and the total volume of the desorption solution at the end of the desorption, respectively; and *q_D_* (mg/g) and *m_D_* (g) are the adsorption capacity of the dye-loaded adsorbents and the weight of the adsorbent, respectively. 

### 2.5. Instrumental Characterization

SEM images were obtained using a S4800 (Hitachi, Tokyo, Japan) microscope. TEM images were obtained using a JEM-2100F (JEOL, Tokyo, Japan) transmission electron microscope. X-ray powder diffraction (XRD) spectra were taken on a D8 advance (Bruker, Billerica, MA, USA) X-ray diffractometer with Cu-Kα radiation. Thermogravimetric analysis (TGA) was carried on a Netzsch STA449F5 analyzer (Netzsch, Selb, Germany) with a heating rate of 10 °C min^−1^ in nitrogen flow. Zeta potential of nanoparticles in aqueous solution was determined by Zetasizer nano ZS (Malvern Instruments Ltd., Malvern, UK) at 25 °C. A TU-1810PC (Persee, Beijing, China) UV-vis spectrometer was utilized for absorbance measurement of dyes in aqueous solutions. The Fourier transform infrared spectroscopy (FT-IR) analysis was performed at room temperature using a Spectrum 100 (Perkin Elemer, Waltham, MA, USA) spectrometer via KBr disk method. Element analysis (EA) was performed on a vario el III (Elementar, Langenselbold, Germany). The magnetic moment was recorded at 300 K on a Lake shore 7404 vibrating-sample magnetometer (VSM) (Lake Shore, Westerville, OH, USA).

## 3. Results and Discussion

### 3.1. Synthesis of Lignin Amine

**Figure 3:**
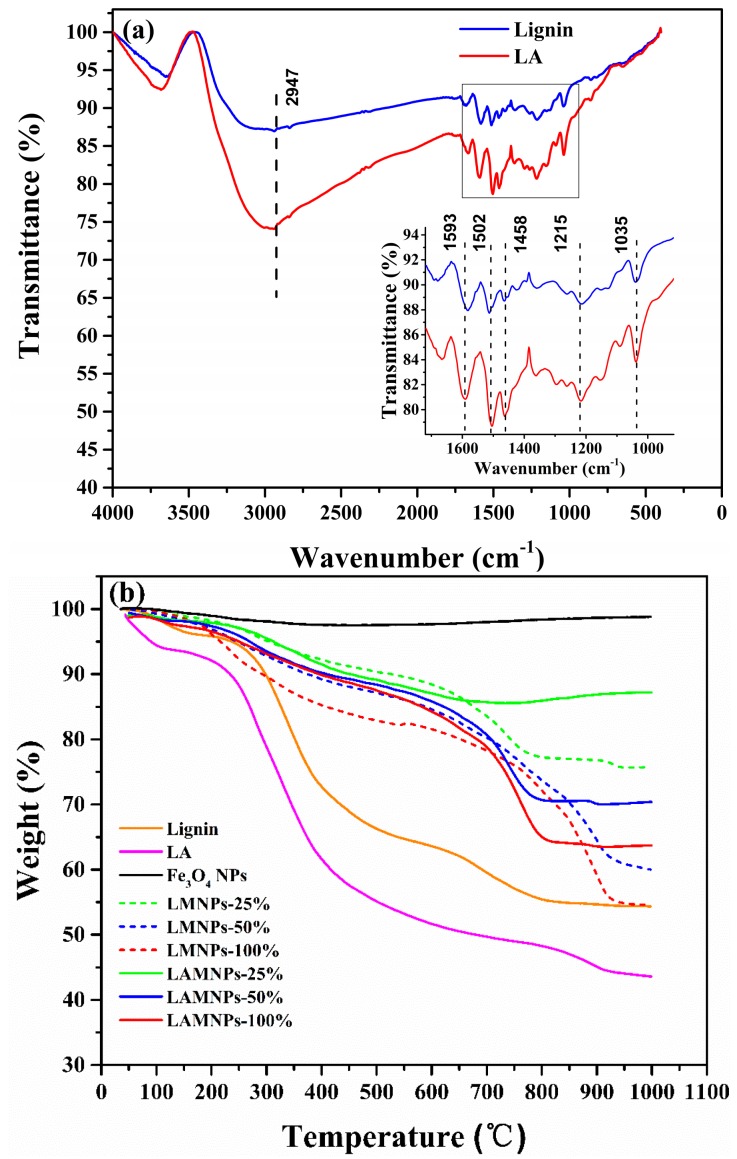
(**a**) FT-IR spectrum of lignin and LA; and (**b**) TGA curves of all samples. The insert of (**a**) is the enlargement of the gray rectangular region.

Figure 3a shows the FT-IR spectra of lignin and lignin amine. Obviously, the adsorption peaks of lignin amine are almost the same as those of lignin. It suggests that the aromatic structure of lignin remained intact after the amination [28]. However, the bending vibration of N–H and C–N bond of DETA were overlapped by the original function groups in lignin. There is only a much stronger absorption peak at 2800–3000 cm^−1^ for LA indicating alkyl group is introduced into lignin structure [32]. 

To further determine the amination result, elemental analysis and TGA were applied to test the lignin and LA. Table 2 shows the elemental analysis results. It is found that the content of nitrogen (N) in LA increase significantly to 6.58% after the amination of lignin (0.51%). However, the C/H atom ratio of LA is evidenced to decrease by 30% compared with lignin. This decrease in C/H atom ratio together with the above increased nitrogen content suggest the introduction of saturated alkyl group into lignin molecular. Meanwhile, the mass fraction of the DETA fragments (DETA%) in LA can be calculated by the following equation:

(4) $$\mathrm{DETA}\%=\frac{{N\%}_{\mathrm{LA}}-{N\%}_{L}}{N_{\mathrm{DETA}}}M_{\mathrm{DETA}}=14.99\%$$

where N%_LA_ and N%_L_ are the content of nitrogen in LA and lignin, respectively; N%_DETA_ is atom weight of nitrogen in DETA; and M_DETA_ is the molecular weight of DETA.

TGA curves of the solid powder samples of lignin and LA under nitrogen atmosphere are shown in Figure 3b. The weight loss of lignin was merely about 4% when it was heated to 180 °C, which was mostly attributed to water loss in lignin. Continuing heating to 900 °C, a further weight loss of about 42% is observed. This weight loss is mainly ascribed to volatilization of the low-molecular-weight lignin fragment due to the lignin decomposition and formation of char. Compared with the lignin, although LA showed similar trend of weight loss, a much more significant weight loss was detected at the corresponding two stages mentioned above. This extra weight loss (10%) is due to the volatilization of free DETA and decomposition of DETA fragments. This is consistent with the mass fraction of DETA fragments in LA (DETA%) calculated from EA results. The above results prove the successful grafting of the DETA fragments to lignin.

### 3.2. Synthesis and Characterization of LMNPs and LAMNPs

The one-step synthesis protocol of LMNPs and LAMNPs was mainly designed based on the combination of co-precipitation and complexation [25,30]. According to previous spectroscopic study [33], the bidentate diphenol ligands (e.g., dopamine) could convert the under-coordinated Fe surfaces sites back to a bulk-like lattice structure with an octahedral geometry for oxygen-coordinated iron. This conversion results in tight binding of bidentate diphenol to iron oxide [34]. Similar to dopamine, lignin is also phenolic compounds. These multiple phenolic groups in lignin make the lignin a multidentate ligand that bind s to iron oxide by coordination. On the one hand, by coordination, lignin or LA can adsorb iron ions by their phenolic groups to act as stabilizer to inhibit aggregation during the nucleation and growth of magnetic nanoparticles (MNPs). On the other hand, the lignin or LA is grafted on the surface of MNPs via coordination bonds between phenolic groups and Fe ion [10].

**Figure 4:**
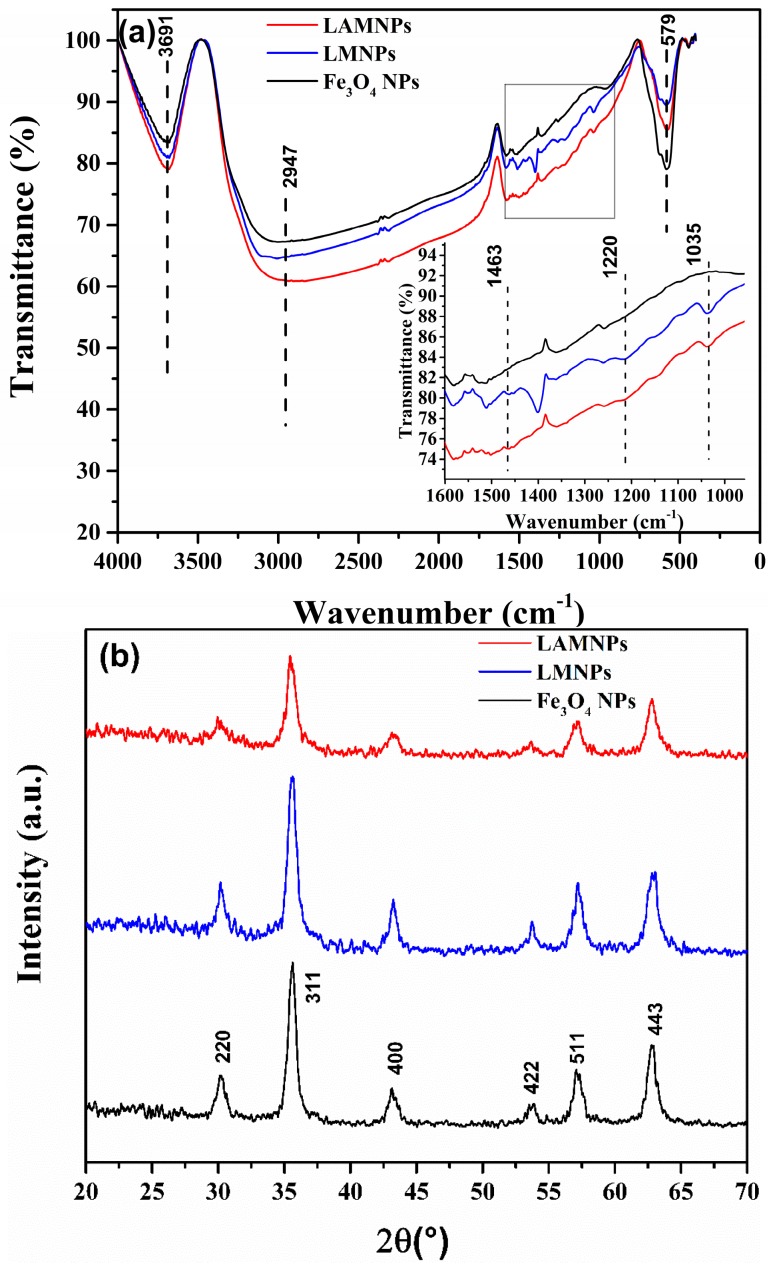
(**a**) FT-IR spectrum of LAMNPs (red), LMNPs (blue) and MNPs (black); and (**b**) XRD patterns of LAMNPs (red), LMNPs (blue) and MNPs (black). The insert in (**a**) is the enlargement of the gray rectangular region.

As shown in the FT-IR spectra in Figure 4a, LMNPs and LAMNPs exhibit a strong Fe–O vibration band at around 580 cm^−1^, indicating the presence of MNPs. Compared to MNPs’ smooth adsorption at the range of 1000–2000 cm^−1^, LMNPs and LAMNPs show many similar absorption peaks (1463 cm^−1^, 1220 cm^−1^, and 1035 cm^−1^) as lignin or LA at this range. It suggests that the surface of MNPs was coated by lignin or LA. The crystalline structure of LMNPs and LAMNPs was identified by XRD (Figure 4b). The peaks at 30.1°, 35.5°, 43.3°, 53.7°, 57.3°, and 62.7° were assigned to the characteristic peaks of Fe_3_O_4_ NPs, demonstrating the crystalline structure of Fe_3_O_4_ was not affected by the lignin and LA. 

The grafting of lignin or lignin amine on the MNPs is further confirmed by the TGA measurement, as shown in Figure 3b. For the neat Fe_3_O_4_ NPs, the weight loss before 1000 °C is observed to be negligible. However, a significant weight loss is obtained for LMNPs and LAMNPs when heating to 1000 °C. There is no doubt that this loss of LMNPs or LAMNPs is mainly contributed to the weight loss of lignin and LA at the surface of the MNPs. In fact, for neat lignin and LA, after the water were vaporized at around 100 °C, they started to decompose easily at 200 °C. Their weight loss experienced a sharp decline during 200–600 °C and slowed down at the range of 600–1000 °C. In contrast, LMNPs and LAMNPs went through a relatively mild decline at the beginning of 200–700 °C while a rapid decline at 700–900 °C. The inverse trend suggested LMNPs and LAMNPs had better thermal stability than neat materials. The above results demonstrate the success of coating lignin or lignin amine on the MNPs.

**Figure 5:**
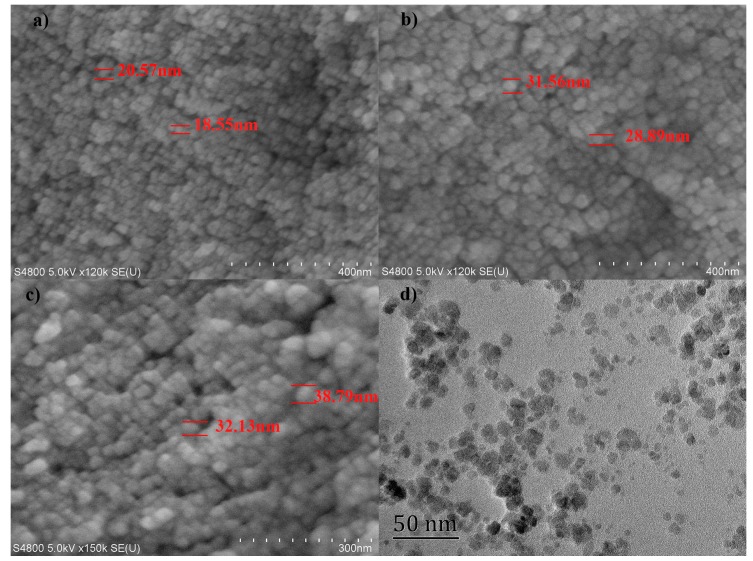
SEM images of: (**a**) LMNPs, 25%; (**b**) LMNPs, 50%; and (**c**) LMNPs, 100%; and TEM image of: (**d**) LMNPs, 50%.

Interestingly, the above grafting of lignin to the MNPs surfaces is further found to be dependent on the ligand content (i.e., lignin and LA contents) added to the reaction system. The difference of ligand content is presented by *f_wt_* (25%, 50%, and 100%), which is the weight feed ratios of lignin or LA to MNPs. Figure 5 and Figure S1 present the morphologies of MNPs with different lignin or LA contents. Obviously, according to the SEM tests, all samples were spherical with narrow size distribution. It is also observed that increasing the lignin dosage facilitates the growth of the LMNPs (from 20 nm at *f_wt_* = 25% to 40 nm at *f_wt_* = 100%). This increase in LMNPs diameter would be ascribed to the accumulated lignin anchored on the surface of MNPs, forming a relatively thick lignin shell. This finding is also supported by the VSM measurements (shown in Table S1 and Figure S2) and TGA results. Increase the lignin or lignin amine content during the reaction, higher drop of the saturation magnetization values and weight loss are observed for both LMNPs and LAMNPs. These results suggest that more lignin molecules are decorated on the surface of MNPs with higher lignin dosage. 

### 3.3. Adsorption of Dyes on LMNPs and LAMNPs

#### 3.3.1. Effects of pH

The solution pH plays an important role in the adsorption of dyes on MNPs, including the adsorption capacity and kinetic, because the ionization of lignin or LA and dye structure are greatly influenced by pH [9]. The difference of ionization ratio of phenolic and amine groups under different pH makes LMNPs and LAMNPs to be pH responsive materials [35]. With amine groups on the solid surface, LAMNPs can be protonated under acid conditions. In contrast, LMNPs are always negatively charged at the pH range of 2–12. These properties allow LMNPs and LAMNPs to possess different adsorption properties to different dyes. 

**Figure 6:**
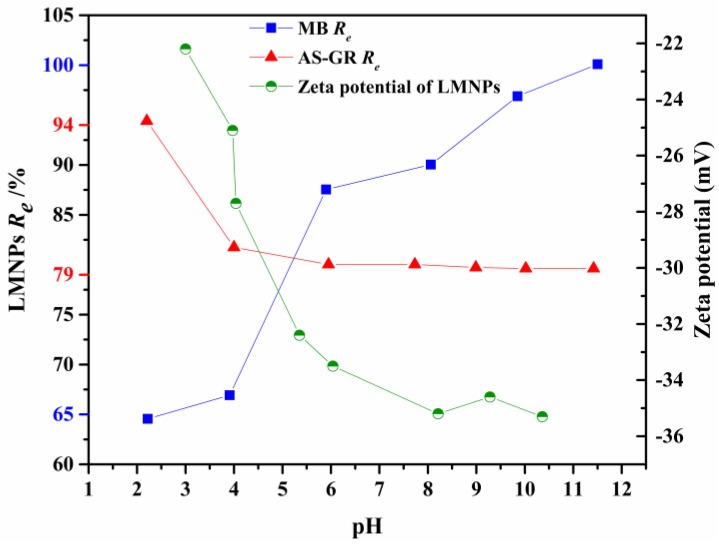
Removal efficiency of cationic and anionic dyes using LMNPs and zeta potential of LMNPs as a function of solution pH (the maximum and minimum removal efficiencies of MB and AS-GR are indicated as blue and red labels, respectively).

**Figure 7:**
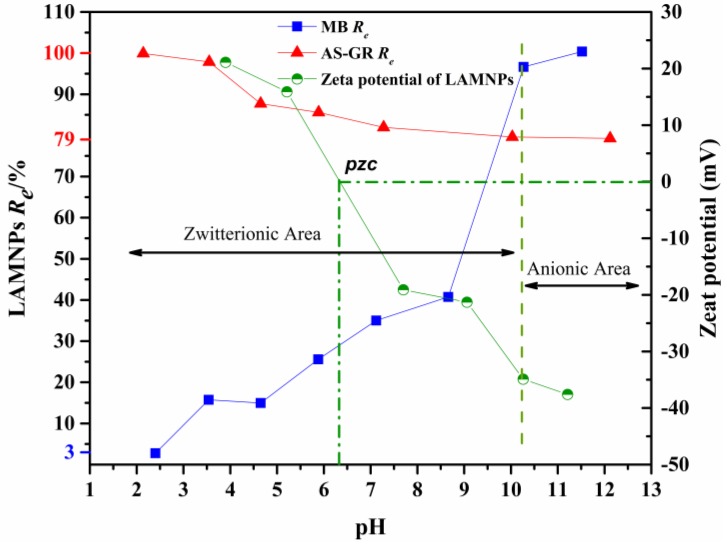
Removal efficiency of cationic and anionic dyes using LAMNPs and zeta potential of LAMNPs as a function of solution pH (the maximum and minimum removal efficiencies of MB and AS-GR are indicated as blue and red labels, respectively).

The adsorption performance of LMNPs and LAMNPs to cationic and anionic dyes were tested under different pH, as shown in Figure 6 and Figure 7. Obviously, the removal of dyes by MNPs adsorption is highly dependent on the solution pH, which illustrates the pH responsive ability of the MNPs. For anionic dyes, both MNPs show good adsorption capability to the AS-GR at acidic conditions (pH = 2). Increasing the pH, the removal of AS-GR from the solution by MNPs adsorption drops sharply by 15–20%, reaching the steady state around pH 10. It should be noted that the LAMNPs perform better (maximum at 100% removal) in adsorbing AS-GR than that of LMNPs in a much wider pH range. However, different things happen to the dye of MB. The best adsorption conditions for MB is in alkaline environment. The increase of MB removal efficiency under alkaline condition is due to the less competitive hydrogen ion in aqueous solution, more negative adsorption sites on LMNPs and the stronger electrostatic attraction force between LMNPs and MB. Both MNPs are found to be able to completely remove the MB dye from the solution above 12. At pH lower than 10, LMNPs present a much better adsorption to MB than that of LAMNPs (up to 60% in difference). Nevertheless, the adsorption of MB on LAMNPs is observed to be much more sensitive to the pH. The removal efficiency of MB in acidic solution is almost 0 at pH 2.5, while it increases (especially at pH 9–10.5) to 100% at pH 12. This adsorption property of LAMNPs to MB is found to be quite different from that of LMNPs. 

Basically, the above differences in MB adsorption between different MNPs could be understood from the following aspects: LMNPs is negatively charged at the pH range of 2–12, but LAMNPs exhibit a point of zero charge (*pzc*). [28] At pH lower than pH_pzc_, the net surface charge becomes positive and at pH higher than pH_pzc_, the net surface charge becomes negative. At the pH near pH_pzc_, there are both protonated amine groups and deprotonated phenolic groups which divide the actual electrostatic state of LAMNPs into two parts, the zwitterionic area and anionic area, as shown in Figure 7 [36]. Because the positive charge exists at zwitterionic area, MB is repulsed by the LAMNPs due to electrostatic repulsion. When the pH comes to anionic area, the electrostatic repulsion disappears, and the *R_e_* of MB for both MNPs becomes the same. This phenomenon indicates that electrostatic force is the main interaction for MB adsorption. Furthermore, it is worth to be noticed that the *R_e_* for anionic AS-GR is above 79%, although there is electrostatic repulsion between LMNPs and AS-GR at the whole range. This indicates that other mechanisms other than electrostatic attraction are operative for AS-GR adsorption on LMNPs. As shown in Table 1, MB and AS-GR is much different not only from the opposite electrostatic charge but also from the structure. MB is a symmetry molecule with heterocyclic nitrogen and two phenylamino groups. It can be protonated on every N atoms of the structure which is polar in aqueous. AS-GR has two sulfonic acid groups on one side of the molecule and can be divided into two parts, the nonpolar aromatic chain and polar end. Based on the difference, this phenomenon may be attributed to the nonpolar aromatic chain of AS-GR that can interact with lignin aromatic rings via π–π stacking while sulfonic acid groups orientated outward forming a monolayer adsorption [37]. This deduction is also proven by the isothermal and kinetic studies below.

#### 3.3.2. Equilibrium Studies

Three isothermal models were used to describe the adsorption isotherms of dyes on the MNPs surface. The linear forms of equations of the tested isotherm models are presented as Equations (5)–(7). The Langmuir isotherm (Equation (5)) assumes a monolayer surface coverage of the dyes on the solid surface structure. The Freundlich isothermal model (Equation (6)) is an empirical model which is applicable for multilayer adsorption. The Tempkin isotherm assumes the decrease of heat of adsorption is linear, when the adsorbate–adsorbent interactions are considered.

(5) $$\frac{C_{e}}{q_{e}}=\frac{C_{e}}{q_{max}}+\frac{1}{K_{L}q_{max}}$$

(6) lnqe=lnKF+1nlnCe

(7) $$q_{e}=\frac{RT}{b}lnA+\frac{RT}{b}lnC_{e}$$

where *q_e_* (mg/g) presents the amount of dyes adsorbed per unit weight of adsorbent when the adsorption reaches equilibrium; *q_max_* (mg/g) denotes the saturation adsorption capacity when complete monolayer coverage is achieved; *K_L_* (L/mg) is the Langmuir constant that relates to the energy of the adsorbent; 1/*n* and *K_F_* are correlated to the relative adsorption intensity and adsorption capacity in Freundlich isotherm, respectively; *b* is the heat of adsorption; and A is the adsorption intensity (binding constant) in Tempkin isotherm; R (J/mol·K) is the gas constant and T (K) is the adsorption temperature.

**Figure 8:**
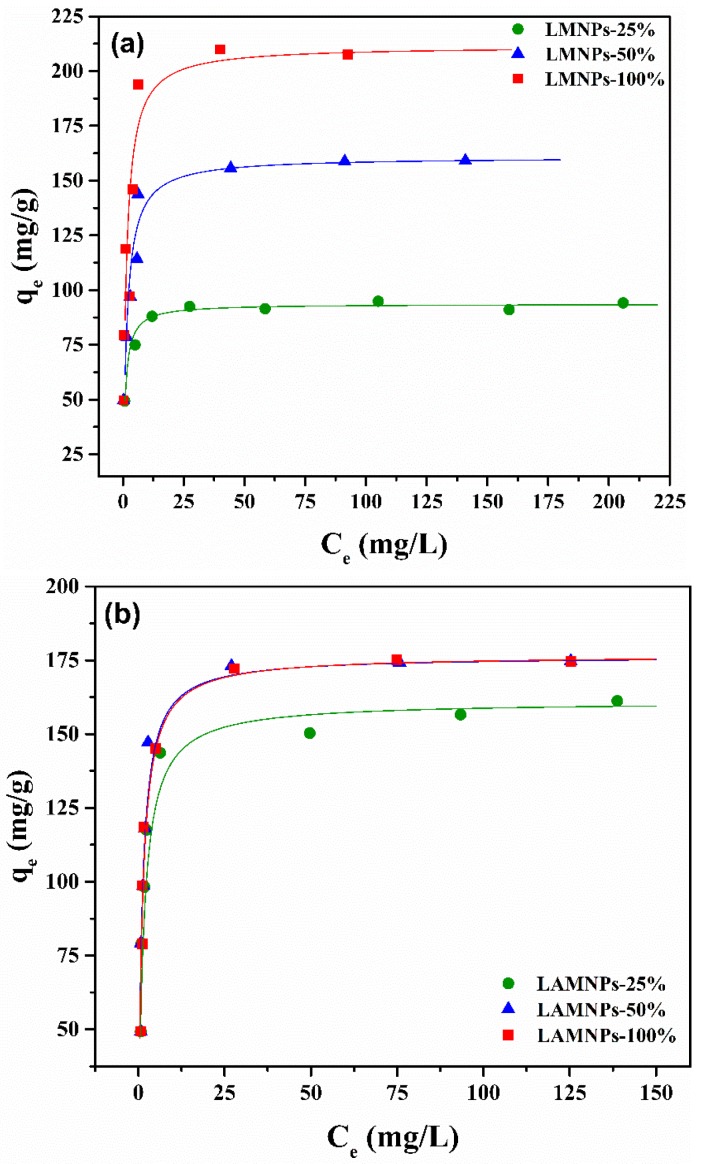
Isotherm experimental data of: (**a**) MB onto LMNPs surfaces and the fitted Langmuir curve at pH 10; and (**b**) AS-GR onto LAMNPs and the fitted Langmuir curve at pH 2.

The linear fitting results of the two isotherms are shown in Table 3 and Figure 8. The Langmuir model best matches the experimental data (*r*^2^ > 0.99, correlation coefficient). This result suggests that the adsorption of dyes onto LMNPs and LAMNPs is a monolayer adsorption. The saturation adsorption capacity (*q_max_*) is found to be positively related to the *f_wt_* due to the larger number of adsorption sites at higher $f_{wt}$. For LMNPs, the maximum adsorption capacity increases from 93.63 to 211.42 mg/g along with the increase of *f_wt_* = 25% to *f_wt_* = 100%. For LAMNPs, at given *f_wt_*, the *q_max_* (176.49 mg/g) is optimized at *f_wt_* = 50%. Further increase of *f_wt_* shows little improvement to the adsorption capacity. Both MNPs show high *q_max_* for dyes, suggesting their great potential for application. Table 4 provides a list of lignin-based adsorbents used for adsorption of different classes of dyes. It is observed that the adsorption capacities of LMNPs and LAMNPs for dyes are much better than those of other lignin based adsorbents shown in the Table 4.

#### 3.3.3. Adsorption Kinetics

**Figure 9:**
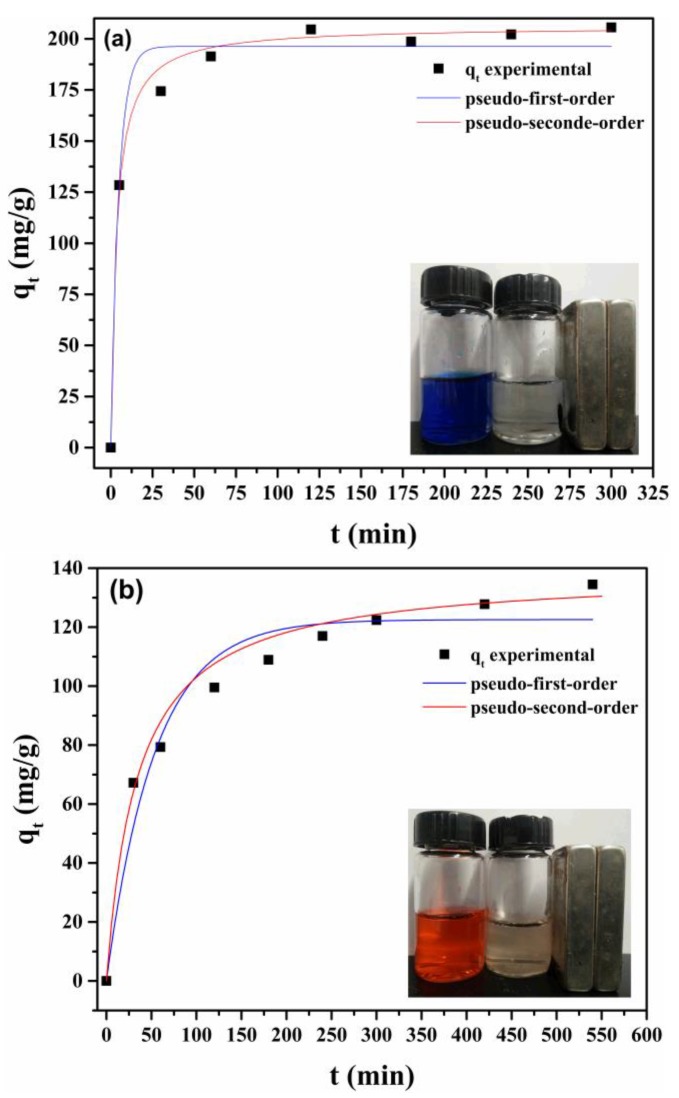
Adsorption kinetic profiles of: (**a**) MB onto LMNPs; and (**b**) AS-GR onto LAMNPs, at the initial concentration of 100 mg/g (for both MB and AS-GR) in 80 mL. The insets are the original dye solution and the separation of the dye-loaded NPs with an external magnetic field.

The adsorption rate is an important parameter for dye removal application. As shown in Figure 9, the time required for maximum removal of different dyes is different. The equilibrium time for MB adsorption onto LMNPs is about 1.5 h. However, more than 10 h is found to be needed for AS-GR adsorption onto LAMNPs, showing a relatively slower adsorption process. This difference on the adsorption rate of these two dyes would be ascribed to the differences in the dye structures. The AS-GR molecules, which possess relatively large molecular volume, are hard to diffuse into the LA shell on the MNPs surface because of the steric hindrance. To attain a stable adsorption on the adsorption sites, AS-GR molecules also need to rearrange their spatial orientation to achieve a maximum overlap area of π–π stacking. MB molecules are smaller and mainly interact with adsorption sites via electrostatic attraction. The absence of steric hindrance also makes the adsorption much quicker. 

The adsorption kinetic data were fitted using two kinetic models (i.e., pseudo-first-order (Equation (9)) and pseudo-second order kinetic models (Equation (8)) to describe the adsorption process.

(8) $$\frac{t}{q_{t}}=\frac{1}{q_{e}}t+\frac{1}{k_{2}q_{e}^{2}}$$

(9) $$ln\left(q_{e}-q_{t}\right)=lnq_{e}-k_{1}t$$

where, $q_{t}$ (mg/g) is the amount of dye adsorbed at the time t (min); $k_{1}$ (min^−1^) and $k_{2}$ (g·mg^−1^·min^−1^) are the pseudo-first-order and pseudo-second-order constant, respectively. $q_{e}$ (mg/g) is the adsorption capacity fitted by kinetic models.

The experimental data and fitting lines of different kinetic models are shown in Figure 9. The corresponding kinetic model parameters as well as the correlation coefficients are given in Table 5. Clearly, for both MNPs and dyes, pseudo-second-order provides the best correlation of experimental data with *r*^2^ over 0.99. This result suggests that the chemical adsorption instead of dye diffusion is the rate-controlling step for the adsorption process [40,41]. 

### 3.4. Mechanism of Adsorption

**Figure 10:**
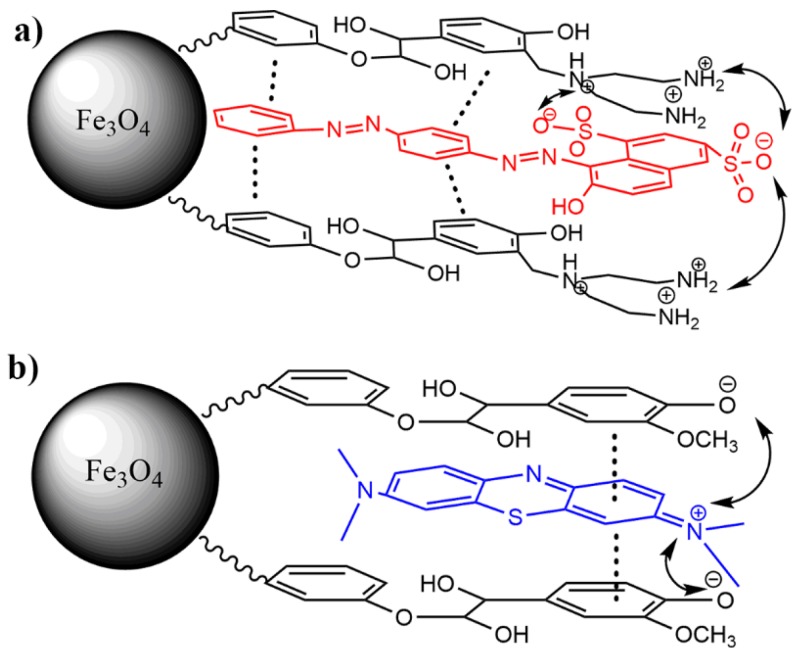
Adsorption profile of: (**a**) AS-GR onto LAMNPs under acid condition; and (**b**) MB onto LMNPs under alkaline condition. The dash and arrowed bonds present π–π stacking and electrostatic attraction interaction, respectively.

According to the above discussion, the adsorption of different dyes onto the dual responsive modified MNPs could be understood as follows: The main interaction force between AS-GR and adsorbents shell is π–π interaction, due to the relatively large nonpolar part of the AS-GR molecule. The protonated cationic amine sites in LAMNPs under acidic condition improve the adsorption efficiency by the electrostatic attraction between the ionized amine groups and AS-GR molecule (Figure 10a). However, with relatively large aromatic structure, AS-GR molecules need more time to diffuse into the adsorption sites and rearrange their orientation to the stable state However, the main interaction force between MB and lignin shell is electrostatic attraction. The positively charged MB molecules are much easier to be adsorbed by the LMNPs with ionized phenolic groups grafted on the surfaces (Figure 10b). When the adsorbent surface possesses cationic sites (LAMNPs), MB cannot be adsorbed by π–π interaction, due to the electrostatic repulsion and lack of nonpolar aromatic part. Comparing to AS-GR, the MB adsorption process is much quicker due to the relatively small molecule volume and global polar structure of MB. 

The mechanism reveals that the adsorption behaviors of LMNPs and LAMNPs are highly dependent on the solution pH and the structure of the dyes. The variation of the pH leads to the change of surface potential and variation in the ionization degree of the polymer surface. The phenolic network of lignin or LA shows an important role in the adsorption mechanism. Anionic dyes with relatively large nonpolar aromatic part tend to be adsorbed by phenolic network due to π–π stacking interaction. Cationic dyes can be adsorbed by electrostatic attraction [5]. This indicates that LMNP and LAMNPs would be an excellent adsorbent for anionic and cationic dyes.

### 3.5. Desorption

The above results prove that the LMNP and LAMNPs would be promising materials for dyes removal from solution due to its high adsorption capacity. Benefiting from the pH responsive property of LMNPs and LAMNPs, the desorption tests are conducted by simply adjusting the pH and monitoring the magnetic field. Results show that the desorption efficiency ($D_{e})$ (Equation (3)) of AS-GR was up to 86%, while $D_{e}$ for MB was relatively low at 46% in 3 h, as shown in Table S2. Further increase in desorption time facilitated a $D_{e}$ of 92% and 50% for AS-GR and MB, respectively. The higher $D_{e}$ of AS-GR is mainly contributed to the electrostatic repulsion between AS-GR and LAMNPs at acidic condition. The high $C_{D}$ value (252.04 mg/L for MB, 265.95 mg/L for AS-GR) at the end of desorption suggested that the adsorbates can be concentrated by adsorption followed by desorption by the two kinds of NPs. These findings suggest that the LMNP and LAMNPs are recyclable nanoparticles through pH response and magnetic response. They would be potential candidates for the recovery of dyes from solution. 

## 4. Conclusions

In this work, an easy and effective method has been proposed to fabricate pH and magnetic dual responsive lignin grafted Fe_3_O_4_ nanoparticles. The obtained MNPs exhibit excellent superparamagnetic property and 2–5 times higher maximum adsorption capacity (211.42 mg/g for MB and 176.49 mg/g for AS-GR) than other lignin based dye adsorbents. The adsorbates on these nanoparticles can also be released with desorption efficiency up to 90% when changing the solution pH. The findings in this paper shed lights on the potential applications of lignin magnetic nano-adsorbents for the remediation of cationic and anionic dye contaminants in water. With the multifunctional property of lignin, the product could also be applied in adsorption of other contaminants, such as heavy metal ions. Further research can fabricate other lignin derivate coated functional materials via this method.

## Figures and Tables

**Table 1:** Information of dyes.

Dye Molecule	Structure	*λ_max_*/nm
MB	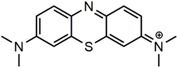	622
AS-GR	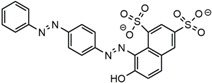	508

**Table 2:** Element analysis results of lignin and LA.

Samples	Element Content wt %			Mole Ratio
	N%	C%	H%	C/H
Lignin	0.51	48.37	4.718	0.85
LA	6.58	43.59	6.174	0.59

**Table 3:** Isotherm parameters for adsorption of MB onto LMNPs and AS-GR onto LAMNPs.

Sample		LMNPs			LAMNPs		
*f_wt_*		25%	50%	100%	25%	50%	100%
Langmuir	*q_max_*(mg/g)	93.63	160.77	211.42	161.03	176.37	176.49
	*K_L_* (L/mg)	1.2404	0.6858	0.7666	0.7017	0.9742	0.9027
	*r*^2^	0.9995	0.9999	0.9991	0.9995	0.9999	0.9999
Tempkin	*b* (J/mol)	345.06	135.14	86.99	142.91	119.31	115.79
	*A*	4443.83	79.87	32.74	129.49	77.61	60.35
	*r*^2^	0.8239	0.8942	0.8585	0.8245	0.8019	0.8435
Freundlich	*K_F_*	59.4764	76.0066	90.8346	80.2623	85.2757	83.2293
	1/*n*	0.1017	0.1804	0.2315	0.1640	0.1808	0.1873
	*r*^2^	0.7918	0.8558	0.8044	0.7117	0.6783	0.7268

**Table 4:** Application of adsorbents based on lignin in the removal of dyes.

Absorbent	Dyes	*q_max_* (mg/g)	Reference
Magnetic lignin hollow microspheres	Methylene blue	31.23	[12]
	Rhodamine B	17.62	
Lignin-chitosan extruded pellets	Methylene blue	36.25	[9]
Acetic acid lignin	Methylene blue	63.3	[38]
Alkali extracted lignin	Methylene blue	121.20	[39]
LMNPs	Methylene blue	211.42	This work
LAMNPs	AS-GR	176.49	This work

**Table 5:** Kinetic model parameters for LMNPs and LAMNPs.

Sample		Pseudo-First-Order			Pseudo-Second-Order		
	*f_wt_*	*q_e_*(mg/g)	*k*_1_ (min^−1^)	*r*^2^	*q_e_*(mg/g)	*k*_2_ (min^−1^)	*r*^2^
LMNPs	100	196.26	0.2105	0.9805	205.76	0.0015	0.9995
LAMNPs	100	122.54	0.0186	0.9574	138.70	0.0002	0.9922

## Supplementary Materials

The following are available online at http://www.mdpi.com/2079-4991/8/3/162/s1, Figure S1: SEM images of LAMNPs; Figure S2: VSM curves of LMNPs and LAMNPs; Table S1: Magnetization value of LMNPs and LAMNPs; Table S2: Desorption efficiency of NPs.
