# Supplementary material for: One-Step Fabrication of Dual Responsive Lignin Coated Fe3O4 Nanoparticles for Efficient Removal of Cationic and Anionic Dyes
Source: Nanomaterials (Basel). 2018 Mar 14;8(3):162. doi: 10.3390/nano8030162 (PMC5869653; doi:10.3390/nano8030162)
Supplement: Supplementary file 1 [file nanomaterials-08-00162-s001.pdf]

nanomaterials

MDPI

One-Step Fabrication of Dual Responsive Lignin Coated Fe_3O_4 Nanoparticles for Efficient Removal of Cationic and Anionic Dyes

Xingang Li [1,2,3], Youyi He [1,2], Hong Sui [1,2,3] and Lin He [1,2,*]

[1] School of Chemical Engineering and Technology, Tianjin University, Tianjin 300072, China; lxg@tju.edu.cn (X.L.); youyihe@tju.edu.cn (Y.H.); suihong@tju.edu.cn (H.S.)
[2] National Engineering Research Centre for Distillation Technology, Tianjin 300072, China
[3] Collaborative Innovation Center of Chemical Science and Engineering, Tianjin 300072, China
* Correspondence: linhe@tju.edu.cn; Tel.: +86-22-27404701

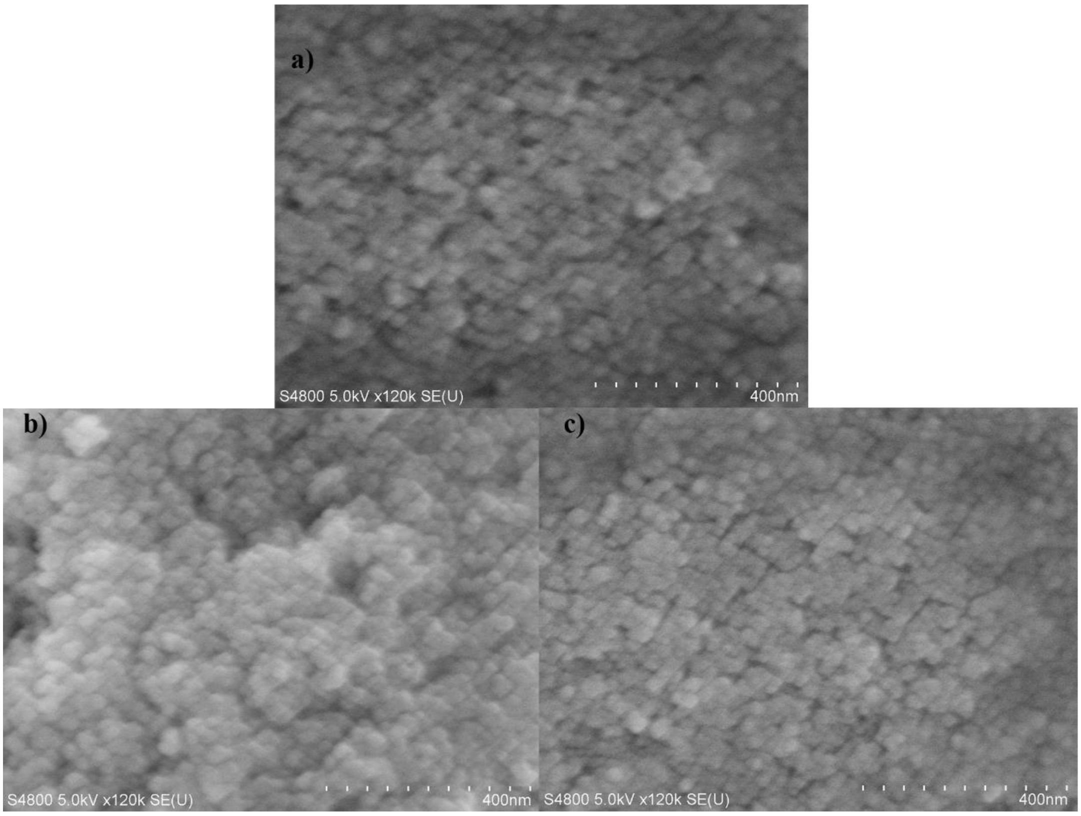

Figure S1. SEM images of (a) LAMNPs-25%, (b) LAMNPs-50%, (c) LAMNPs-100%.

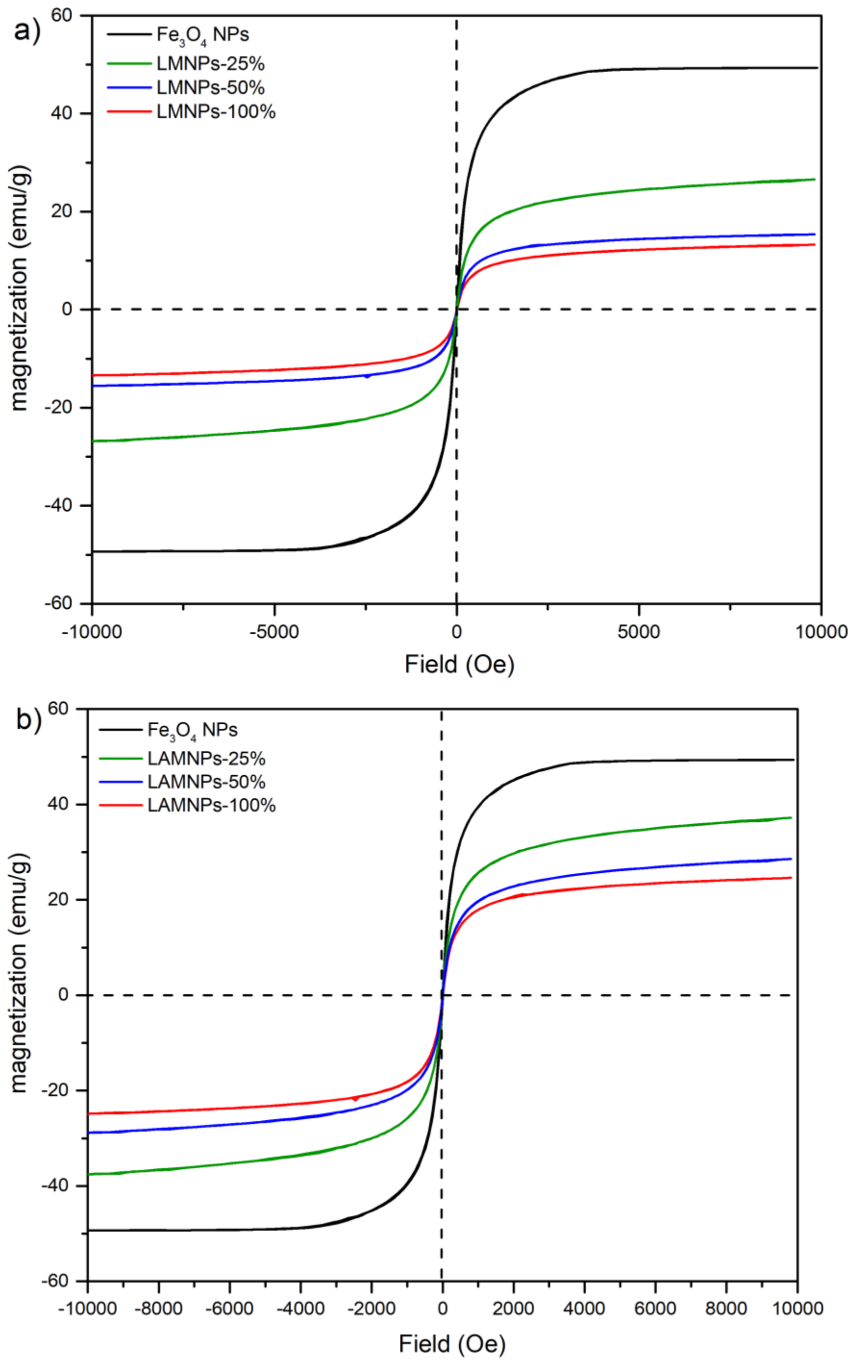

Figure S2 VSM magnetization curves of Fe3O4 NPs, (a) LMNPs and (b) LAMNPs.

Table S1. Magnetization value of LMNPs and LAMNPs.

Samples	f_{wt}	Magnetization (emu/g)
Fe_3O_4 NPs	--	49.33
	25%	26.56
LMNPs	50%	15.38
	100%	13.28
	25%	37.19
LAMNPs	50%	28.55
	100%	24.61

Table S2. Desorption efficiency at different time intervals.

Samples	D_e%		q_D (mg/g)	C_D (mg/L) at 12h
	3h	12h		
MB loaded LMNPs-100%	46.27%	50.02%	251.92	252.04
AS-GR loaded LAMNPs-100%	86.45%	92.28%	144.10	265.95
